# Determining the Public Health Impact of Climate Change: A National Study Using a Health Impact Assessment Approach in Wales

**DOI:** 10.3389/ijph.2024.1606972

**Published:** 2024-04-24

**Authors:** Liz Green, Kathryn Ashton, Nerys Edmonds, Michael Fletcher, Sumina Azam, Karen Hughes, Phil Wheater, Mark A. Bellis

**Affiliations:** ^1^ Public Health Wales NHS Trust, Cardiff, United Kingdom; ^2^ Department of Geographical and Environmental Science, Manchester Metropolitan University, Manchester, United Kingdom

**Keywords:** climate change, health impact assessment, population groups, environment, health and wellbeing

## Abstract

**Objective:** Climate change is recognised as the biggest threat to global health of the 21st century and impacts on health and wellbeing through a range of factors. Due to this, the need to take action in order to protect population health and wellbeing is becoming ever more urgent.

**Methods:** In 2019, Public Health Wales carried out a comprehensive mixed-method Health Impact Assessment (HIA) of climate change. Unlike other risk assessments, it appraised the potential impact of climate change on health and inequalities in Wales through participatory workshops, stakeholder consultations, systematic literature reviews and case studies.

**Results:** The HIA findings indicate potential impacts across the wider determinants of health and wellbeing. For example, air quality, excess heat/cold, flooding, economic productivity, infrastructure, and community resilience. A range of impacts were identified across population groups, settings, and geographical areas.

**Conclusion:** These findings can inform decision-makers to prepare for climate change plans and policies using an evidence-informed approach. The work has demonstrated the value of a HIA approach by mobilising a range of evidence through a transparent process, resulting in transferrable learning for others.

## Introduction

Climate change has been recognised as the biggest threat to global health of the 21st century, contributing to widening health inequalities [1]. The policies currently in place for reducing emissions with no additional action are projected to result in a global warming of 2.8°C over the 21st century [2]. However, to prevent further global warming, it is clear emissions need to be reduced, which requires a rapid transformation of societies and economies [3]. This fundamental shift is required in political, economic and personal action to protect and promote the health and wellbeing of the global population [4, 5].

In response to rapid increases in extreme weather and public concern, governments internationally have declared a climate emergency [6]. A climate emergency was declared in Wales, United Kingdom in 2019 by the Welsh Government [7] who appointed the first Minister for Climate Change in 2021 [8]. Wales is a country in the United Kingdom which borders with England and the Irish and Celtic seas, with a population of around 3.1 million people. Increasingly frequent extreme weather events have occurred in Wales in recent years [9], impacting infrastructure, services, and the physical and mental health of the population [10]. Wales has a strong record for assessing the public health implications of policies and events [10–12]. This is further supported by the enabling environment to facilitate sustainable development, address climate change and create a more resilient, healthier Wales. The Wellbeing of Future Generations (Wales) Act (2015) [13] is an example. This legislation focuses on sustainable development, making overt connections to health, equity and determinants of health such as resilient communities and global responsibility to meet the needs of Wales today without compromising the future needs of the country. National recognition of climate change as a cross-cutting policy crisis, highlights the importance of “Health in All Policies” approaches (HiAP) [14].

To further understand the health and wellbeing impacts of climate change on the Welsh population, the national public health agency in Wales, Public Health Wales, carried out a Health Impact Assessment (HIA). HIA is a process that considers the impact of a policy, plan or scenario on the health and wellbeing of any specific populations who may be particularly affected [12, 15]. It is commonly defined as “a combination of procedures, methods and tools by which a policy, plan or project may be judged as to its potential effects on the health of a population, and the distribution of those effects within a population” [16]. HIAs can help to identify evidence-based actions and mitigation to diminish or remove negative impacts and maximize positive ones [12]. As an internationally established process and public health tool, HIA is both systematic and flexible helping to raise awareness and consideration of health, wellbeing and inequalities [17]. HIA is also strongly linked to the HiAP approach and is recognised by the WHO as an important tool to enable collaboration and for health and wellbeing to be considered in all policy sectors [14].

Whilst the environmental and economic impacts of climate change are well documented [1, 2, 18], there is less evidence demonstrating the wider public health and wellbeing impacts on specific population groups beyond the displacement of people [18]. A recent synthesis of systematic reviews of the health effects of climate change [19] concluded that most studies focused on the “meteorological impacts of climate change on adverse physical health outcomes” and that “future studies could fill knowledge gaps by exploring other climate-related impacts and broader psychosocial health outcomes.” In addition, the authors highlight a need to “unpack the ‘what, how, and where’ of these effects.” This novel study aims to contribute to this gap by outlining the process and findings from a national level HIA in Wales, which focused on the “what, how and where” of health and wellbeing impacts of climate change as well as the cumulative impacts on specific population groups. It also provides policy focused recommendations for the future.

This assessment is unique in its approach to use the HIA process to identify the populations who may be potentially majorly affected by climate change either positively or negatively, alongside the potential impacts on the determinants of health and wellbeing. It aims to provide relevant and transferrable learning for international, national and cross-sector policymakers and practitioners who have an interest in environmental and sustainability issues, climate change or those who may wish to carry out HIAs on major events and challenges. The data and evidence collected as part of the HIA process can also aid organisations with adaptation planning through enabling prioritisation of impacts and the framing of key messages on climate change. It will inform public health and climate policymakers, public bodies, third and private sector agencies about the potential positive and negative impact on people’s health and wellbeing and identify any structural inequality, which may emerge from climate change and extreme weather events.

## Methods

HIA as practiced by the Wales Health Impact Assessment Support Unit (WHIASU) at Public Health Wales is based on the WHO definition of health and follows a systematic yet flexible evidence-based process [17]. It assesses the impact of policies, plans or projects through the lens of the wider determinants of health [20]. The HIA process follows a mixed methods approach, using both quantitative and qualitative methods to consider the potential positive and negative (including unintended) impacts on population groups and health equity. This is achieved by using secondary evidence, but also carrying out primary stakeholder engagement with organisations, public bodies, and communities who could be impacted by a policy, plan or project. The HIA process uses two checklists [21] that focus on the wider determinants of health and population groups to ensure comprehensive potential impacts are considered.

In 2019, a comprehensive mixed method HIA of climate change in Wales was started and conducted over a 6-month period. However, the COVID-19 pandemic led to its suspension at the appraisal stage and the HIA was restarted and updated from mid-2022 [22]. The internationally recognised five-step process of a HIA was followed [17].

### Screening and Scoping

A multi-disciplinary, cross-sector Strategic Advisory Group (SAG) was established to oversee the HIA alongside Public Health Wales’ governance processes. This consisted of representatives from a wide range of public bodies and groups including national and local government, the national environment agency, local health services providers, academia and a third sector sustainability focused group (Renew Wales, n.d.). The SAG also provided guidance on the HIA process, and feedback on the final report. In addition, an internal Working Group was established. Using the WHIASU Wider Determinants checklist and the Population Groups checklists as a guide [21], the screening process identified a range of potential determinants of health and wellbeing and population groups, which could be affected by climate change. The scope of the HIA was also determined, with the geographical boundary of the HIA defined as Wales. The scoping allowed for the parameters and timeframe of the project to be set and outlined the resources required to undertake the assessment.

The HIA also utilised the established conceptual framework for assessing socio-spatial vulnerability and climate disadvantage [23]. This enabled identification of vulnerability in different population groups in relation to climate change, and relationships between personal factors linked to vulnerability, the social determinants of health, other structural factors and inequalities. A research protocol was developed for a rapid review of grey and academic peer-reviewed evidence. Academic literature was identified via ProQuest and grey literature via Google Advanced Search. Evidence was included if published in the English language in the last 10 years and focussed on the key topic of climate change, including the wider determinants of health and key population groups. All results were screened and reviewed by two independent researchers. If deemed eligible for inclusion, evidence was reviewed, and relevant information extracted into an extraction table for use in the evidence appraisal process. A community health profile was developed using quantitative health intelligence data obtained from robust evidence sources in Wales. This included the National Survey for Wales [24] and the Welsh Index of Multiple Deprivation [25].

Thirty-three stakeholders were invited to participate in semi-structured qualitative interviews, with 25 interviewed across 19 interviews. Ninety stakeholders were invited to participate in two cross-sector, multidisciplinary participatory workshops. The purpose of the workshops were to gather qualitative evidence and knowledge from key stakeholders. Participating stakeholders, identified through purposeful sampling based on the findings from the screening stage, included representatives from a wide range of public bodies, frontline services, academia and the third sector (for example, health and social care, housing, emergency services, planning and community representatives). In total, 33 stakeholders (approximately 30% of those invited) participated in the workshops, which were independently facilitated by members of the WHIASU team. The HIA team felt that participants were representative of all key stakeholder groups, despite only 33 out of the 90 attending. No formal ethical approval was required to undertake this assessment, and all participants agreed to findings being reported anonymously and consent was taken before participation. Workshop participants also completed a feedback and evaluation form. The workshops and interviews were transcribed, and transcripts thematically analysed by members of the HIA team.

### Evidence Appraisal

All evidence gathered at the screening and scoping stage was collated and synthesised to assess and characterise the positive and negative impacts, and form a picture of the scale, scope and duration of these. The impact was considered for both the identified population groups and the wider determinants of health. This process has been used in previous high-profile HIAs and has been published elsewhere [22, 26]. The identified impacts were also classified into the categories defined in Table 1.

**TABLE 1:** Characterization of impact (Wales. 2020).

Intensity	
Minimal	Of a minimum amount, quantity or degree
Moderate	Average in intensity, quality or degree
Major	Significant in intensity, quality or extent
Duration	
Short term	Impact seen in 0–1 year
Medium term	Impact seen in 1–5 years
Long term	Impact seen in over 5 years
Likelihood	
Possible	Plausible, but with limited evidence to support
Probable	Direct evidence but from limited sources
Confirmed	Strong direct evidence from a wide range of sources that an impact has already happened or will happen

### Reporting

A full HIA report was developed including recommendations for action, which was reviewed by the SAG and approved by the Executive Team of Public Health Wales. It was also subject to a stringent quality assurance process using the WHIASU Quality Assurance Review Framework [27]. The final report was disseminated via a network of key stakeholders compiled throughout the HIA process and via relevant specialist public health and climate change networks including the Faculty of Public Health Special Interest Group on Sustainable Development, the Welsh Government climate emergency programme and Green Health Wales.

### Monitoring and Evaluation, Including Review and Reflection

Findings of the HIA were shared to inform the development of a monitoring system of health impacts arising from climate change in Wales via public health surveillance. Formative evaluation and learning [28] is an important part of HIA in Wales [12, 26]. A review and reflection session was held by the HIA team after publication to capture the learning from the HIA, what did and did not work, and the impact the process had on other workstreams, policies and decisions to date. Notes were also captured by the lead authors throughout the process of carrying out the HIA.

## Results

The HIA findings indicate significant positive and/or negative impacts across the wider determinants of health and mental wellbeing, population groups, settings, and geographical areas. The impacts are both confirmed (current) and potential, and identified as short, medium or long-term in duration (Table 2). The evidence highlighted that climate change is complex and an important determinant of wellbeing, having potential major, multifaceted, co-occurring and inequitable impacts. For example, climate change was found to have an impact on: nutrition and food security; community resilience and cohesion; mental health and wellbeing (i.e., a person’s condition with regard to their psychological and emotional wellbeing). The determinants affected and additional areas were identified as needing more evidence to highlight impacts are depicted in Table 2.

**TABLE 2:** Determinants of health and wellbeing (Wales. 2023).

	Negative/Unintended negative impacts^a^			Positive/Opportunities		
Health determinant	Intensity^b^	Likelihood^c^	Duration^d^	Intensity^b^	Likelihood^c^	Duration^d^
Food utilisation: healthy eating	Unknown	Unknown	Unknown	No evidence	Possible	Short/long-term
Food availability: production and security	Major	Probable	Short/long-term	No evidence	Possible	Short/long-term
Food accessibility: cost of food	Major	Probable	Short/long-term	Unknown	Unknown	Unknown
Nutritional content of food	Major	Probable	Short/long-term	No evidence	Possible	No evidence
Food Borne Disease	Moderate	Possible	Unknown	Unknown	Unknown	Unknown
Physical activity and outdoor leisure activity	Moderate	Possible/probable	Short/medium term	No evidence	Possible	Short/long-term
Alcohol and substance misuse	No evidence	Possible	Unknown	Unknown	Unknown	Unknown
Community resilience and cohesion	Moderate/major	Possible/probable	Short/long-term	Moderate/major	Probable	Short/long-term
Population displacement, mobility and migration	Major	Probable/confirmed	Short/long-term	No evidence	Possible	No evidence
Violence	No evidence	Possible	No evidence	Unknown	Unknown	Unknown
Family and intergenerational relationships	No evidence	Possible	Short/long-term	Unknown	Unknown	Unknown
Mental wellbeing	Major	Probable	Short/long-term	No evidence	Possible	Short/long-term
Social isolation	Unknown	Possible	Short/long-term	Unknown	Unknown	Unknown
Mental disorder	Major	Probable/confirmed	Short/long-term	Unknown	Unknown	Unknown
Suicide	No evidence	Possible	No evidence	Unknown	Unknown	Unknown
Housing	Moderate/major	Probable/confirmed	Short/long-term	Moderate	Possible	Short/long-term
Air Quality (Outdoors)	Moderate	Possible	Short/long-term	Moderate	Possible	Short/long-term
Flooding	Major	Confirmed	Short/long-term	Unknown	Unknown	Unknown
Higher temperature and extreme heat	Major	Probable	Short/long-term	Moderate	Probable	Medium/long-term
Water Supply	Moderate/major	Probable/confirmed	Short/long-term	Unknown	Unknown	Unknown
Water Quality	Moderate/major	Confirmed	Short/long-term	Unknown	Unknown	Unknown
Natural environment and biodiversity	Major	Confirmed	Short/long-term	Moderate	Possible	Short/long-term
Landslides and coal tip	Major	Possible/probable	Short/long-term	Unknown	Unknown	Unknown
Wildfires	Major	Probable	Short/long-term	Unknown	Unknown	Unknown
Vector borne disease	Moderate/major	Possible -	Short/long-term	Unknown	Unknown	Unknown
Working Conditions	Moderate/major	Probable	Short/long-term	No evidence	Possible	No evidence
Economic Development and skills	Major	Confirmed	Short/long-term	Moderate/major	Possible	Short/long-term
Access to health and care services	Moderate/major	Probable/confirmed	Short/long-term	Moderate	Possible	Short/long-term
Education	Moderate/major	Probable	Short/long-term	Moderate	Possible	Short/long-term
Transport	Moderate/major	Confirmed	Short/long-term	Moderate	Probable	Short/long-term
Infrastructure	Major	Confirmed	Short/long-term	Unknown	Unknown	Unknown

Unknown—no or lack of evidence. This does not mean that there is no impact and therefore is a research gap.

^a^ Unintended negative impacts are impacts that are considered to diminish health status.

^b^ Intensity: i.e., minimal; moderate; major.

^c^ Likelihood: i.e., probable; possible; confirmed.

^d^ Duration: i.e., short/medium/long-term.

### Population Groups

This HIA demonstrates that the whole population in Wales is impacted by climate change, with particular population groups being more affected positively, negatively or both (Table 3). For example, *older adults* aged 65 and over accounted for 21% of Wales’ total population in 2021, and this proportion is projected to increase by around 16% by 2030 [29]. The population aged over 75 is also projected to increase from 9% of the population in 2021 to around 13% in 2030. Older adults are more vulnerable to health impacts from extreme heat, poorer air quality and extreme weather, but would be positively impacted by the potential for warmer winters. *Limiting longstanding illness* affected a third (33%) of adults in Wales in 2021/22 [30] and people in Wales live on average for around 18 years not in good health. People living with a range of health conditions were found to be more vulnerable to climate change related health impacts including higher temperatures, flooding and reduced air quality, with no positive impacts identified.

**TABLE 3:** Population groups identified as particularly affected by climate change in Wales (Wales. 2023).

	Negative/Unintended negative impacts^a^			Positive/Opportunities		
Population group	Intensity^b^	Likelihood^c^	Duration^d^	Intensity^b^	Likelihood^c^	Duration^d^
Babies, children and young people	Major	Probable/confirmed	Short/long-term	No evidence	Possible	Short/medium term
Older adults	Major	Probable/confirmed	Short/long-term	Unknown	Unknown	Unknown
Women	No evidence	Probable	No evidence	No evidence	Possible	No evidence
Pregnant women	Moderate	Probable	Short/long-term	Unknown	Unknown	Unknown
Men	Moderate	Probable/possible	Short/long-term	No evidence	Possible	Short/long-term
People who are displaced including refugee and asylum seekers	Major	Probable	Short/long-term	Unknown	Unknown	Unknown
People with long-term health conditions and/or disabilities	Moderate/major	Probable	Short/long-term	Moderate/major	Probable	Short/long-term
People who are homeless	Major	Probable/confirmed	Short/long-term	No evidence	Possible	No evidence
Ethnic minority groups	Moderate	Probable	Short/long-term	Unknown	Unknown	Unknown
People who are new to an area	Moderate	Probable	Short/long-term	Unknown	Unknown	Unknown
Low-income groups	Major	Probable/confirmed	Short/long-term	No evidence	Possible	Short/long-term
Occupational groups including outdoor workers; manufacturing; transport workers; and health and social care staff and emergency services	Moderate	Probable	Short/long-term	Unknown	Unknown	Unknown
Coastal areas	Major	Confirmed in some areas	Short/long-term	Unknown	Unknown	Unknown
Flood risk areas	Major	Confirmed	Short/long-term	Unknown	Unknown	Unknown
Former and current industrial areas	Moderate/major	Possible	Short/long-term	Moderate	Possible	Short/long-term
Urban areas	Major	Probable/confirmed	Short/long-term	Moderate	Possible	Short/long-term
Rural communities	Major	Probable	Short/long-term	Unknown	Unknown	Unknown
Areas of multiple disadvantage	Moderate/major	Probable	Short/long-term	Moderate	Probable	Medium/long-term

^a^ Unintended negative impacts are impacts that are considered to diminish health status.

^b^ Intensity: i.e., minimal; moderate; major.

^c^ Likelihood: i.e., probable; possible; confirmed.

^d^ Duration: i.e., short/medium/long-term.

Occupational groups such as those working in education, social care and emergency services, those who worked outdoors and those who worked in education and transport were identified to be potentially impacted by climate change. Over 40% of the workforce in Wales work in jobs identified as being more exposed to the health and wellbeing impacts of climate change. Coastal Communities were identified to be majorly affected with a significant proportion of the population potentially exposed to the health and wellbeing impacts of sea level rise, coastal erosion and flooding [31].

In addition, the HIA identified that the way that climate adaptation policy and interventions are designed, communicated and implemented also incur impacts for health and wellbeing beyond the meteorological effects. A range of policy responses, action and mitigations to tackle climate change and its effect on health and wellbeing were identified (Box 1).

Box 1 **Identified policy responses, actions and mitigation to tackle climate change (Wales. 2023).**

• Enhancing mitigation and adaptive capacity
• Enhancing risk communication and education
• Enhancing public involvement and engagement in adaptation policy
• Enhancing the integration of health and wellbeing into adaptation planning across sectors
• Investing in co-benefits for health.

## Discussion

The HIA outlined in this paper examined the health, wellbeing and equity impacts of climate change on the population of a small, high income, temperate country, and identified the potential and actual health impacts for a range of different population groups and key determinants of health. Using a well-established HIA process, a wide range of evidence was appraised, and key cross-sectoral, multi-disciplinary stakeholders participated to ensure a rich level of both positive and negative impacts were captured along with a wide range of perspectives. It has provided key recommendations to inform current planning and future policy decisions and actions to mitigate for the outcomes of climate change. Stakeholder knowledge and engagement is an important building block of the impact assessment process [32–34]. Whilst peer reviewed papers and data are weighted more heavily than the qualitative evidence, the insight and the knowledge captured through the extensive stakeholder involvement has proved valuable to provide local contextual knowledge and a better understanding of the climate change, health, demographic, topographic and policy landscape in Wales and has strengthened the reach of the HIA. The findings of the HIA have been mobilised within Wales, both within Public Health Wales and across public bodies, including Welsh Government. Public Health Wales have a new strategic priority of “Tackling the public health effects of climate change” [35], and the HIA findings fed directly into the Welsh Government New National Adaptation/Resilience Plan which is due to be launched in 2024.This HIA has demonstrated it is imperative that impacts are fully identified and understood so that the “what, how and where” of climate change can inform adaptation planning. The value of HIA in identifying vulnerability and inequalities arising from climate change has been highlighted previously [36–38]. The granular approach of the HIA using the two checklists as guides makes it particularly useful as it can enable a ‘deep dive’ into key topics and identify the vulnerable subgroups or determinants that are particularly relevant to specific regions or countries.

A further value of the HIA was that it enabled the application and contextualisation of the international evidence on health impacts of climate change to a specific country level population, demonstrating the proximal rather than distal nature of these impacts to policymakers. High levels of the population believe climate change is something with consequences elsewhere (or at least that the major ones are abroad, for example, wildfires in Australia). Media coverage often shows these big events elsewhere and it is easy to get the impression that Wales, for example, is relatively untouched except for flooding or storms. Another benefit of a nation carrying out their own HIA is that the process can lead to professional stakeholders and others locally/nationally (for example, third sector or community leads) to challenge this concept, recognise that climate change is already significantly impacting issues in the nation itself, and lead to the realisation that they need to take action on those issues.

The findings of this HIA aligns with previous research, which states that the impacts of climate change on health and wellbeing are not distributed equally [19, 23, 39] and that, and, specifically for the United Kingdom, “opportunities for adaptation are unlikely to be unevenly distributed across the UK population” [40]. The HIA also identified significant cumulative impacts for different population groups, highlighting the need for population and place-based vulnerability to be integrated into adaptation and resilience planning as much as sectoral and service-based risks. The HIA highlighted that climate change does not exist in a vacuum, impacting on various determinants of health in a complex systems way. As identified in this HIA and in previous research [41, 42], there are range of action and mitigations that can be implemented by a wide range of policies and actors. It also highlights that there can be co-benefits for both health and the environment from mitigation and adaptation, for example, more active travel leading to less car emissions and cleaner air for those with respiratory conditions [22]. However, caution is also needed as an intervention that may have benefits to health, the environment, or a specific population group could also have unintended negative impacts for others, such as the economy [42, 43].

These results contribute to the growing body of evidence that climate change adaptation is not keeping pace with climate change [44–47]. An enhanced approach and investment is needed in policy and practice across different sectors. For example, through investing in action guided by enhanced, robust public health surveillance of the impact of climate change on health and wellbeing, and involving the public via democratic decision-making processes and policymaking. Investing in co-benefits for health [48], such as investing in strategies and climate resilient infrastructure to increase physical activity and active travel, will contribute to tackling climate change.

It is recognised that there are limitations to this study. It is noted that some impacts are predictive or potential, due to the ever-changing nature of climate change, for which the future is unknown [49]. However, this HIA has been useful in identifying potential impacts and providing recommendations for action by the appraisal of relevant robust evidence and presenting it in a coherent format (Tables 1, 2). Although this HIA is based solely on the Welsh context, many impacts may be generalizable across different contexts and nations. Finally, although the impacts identified within this HIA were based on an extensive review of the available literature at the time, the evidence base on climate change and climate science is rapidly developing [50]. As a result, it is necessary for the findings from this assessment to be reviewed and updated.

The results from this HIA can be used as the basis for future research. It has demonstrated how climate change is not an isolated phenomenon. Therefore, it is essential that further investigation is conducted in several areas to strengthen the evidence base to address interconnected challenges. These include, for example, identifying areas of potential increased health service demand arising from climate change, and on effective approaches to manage and mitigate the impacts of flooding. This study also found that further research is required to identify the impacts of extreme weather on mental health and wellbeing in particular population groups. Significant gaps in the literature were also recognised in relation to the Welsh context, for example, the health impacts of extreme heat, drought and vector borne disease, and also implications for mental health and wellbeing, violence, alcohol and substance misuse, and family and intergenerational relationships. Moreover, this work has demonstrated the applicability and suitability of implementing HIA to capture the holistic impacts of climate change [36]. Further research could build on this to make results more applicable to local populations and different contexts. This can be achieved by mirroring the HIA process undertaken in Wales, and engaging with stakeholders relevant to the context in question.

### Conclusion

This study depicts the major potential impacts and opportunities for health and wellbeing which can emerge from climate change at a country level, using Wales as an example. The findings have been beneficial to inform decision-makers developing plans and policies on climate change using an evidence-informed approach. The work has demonstrated the value of an HIA approach for addressing the impacts of climate change on health and wellbeing, by engaging stakeholders and mobilising a range of evidence through a transparent process, resulting in transferrable learning for others.
